# Exploring effects of different male parent crossings on sheep muscles and related regulatory genes using mRNA-Seq

**DOI:** 10.5713/ab.21.0463

**Published:** 2022-01-05

**Authors:** Jinping Shi, Quanwei Zhang, Yali Song, Zhaomin Lei, Lingjuan Fu, Shuru Cheng

**Affiliations:** 1 College of Animal Science and Technology, Gansu Agricultural University, Lanzhou 730070, China; 2 College of Life Science and Biotechnology, Gansu Agricultural University, Lanzhou 730070, China

**Keywords:** Cross, Male Parent, Meat quality, Regulatory Gene, Sheep

## Abstract

**Objective:**

With improvements in living standards and increase in global population, the demand for meat products has been increasing; improved meat production from livestock could effectively meet this demand. In this study, we examined the differences in the muscle traits of different male crossbred sheep and attempted to identify key genes that regulate these traits.

**Methods:**

Dubo sheep×small-tailed Han sheep (DP×STH) and Suffolk×small-tailed Han sheep (SFK×STH) were selected to determine meat quality and production performance by Masson staining. Transcriptome sequencing and bioinformatic analysis were performed to identify differentially expressed genes (DEGs) related to meat quality. The presence of DEGs was confirmed by real-time polymerase chain reaction.

**Results:**

The production performance of SFK×STH sheep was better than that of DP×STH sheep, but the meat quality of DP×STH sheep was better than that of SFK×STH sheep. The muscle fiber diameter of DP×STH sheep was smaller than that of SFK×STH sheep. Twenty-two DEGs were identified. Among them, four gene ontology terms were related to muscle traits, and three DEGs were related to muscle or muscle fibers. There were no significant differences in the number of single nucleotide mutations and mutation sites in the different male parent cross combinations.

**Conclusion:**

This study provides genetic resources for future sheep muscle development and cross-breeding research.

## INTRODUCTION

Lamb meat has a high protein content and a unique taste; according to the traditional Chinese medicine, lamb has health-promoting functions. Therefore, lamb is popular among Chinese diners, and its consumption is increasing annually. However, with the improvements in general living standards, people are no longer satisfied with low-quality lambs. In this milieu, producing sheep that can adapt to the Chinese environment and have a better meat quality has become a focus of Chinese animal husbandry research. China has abundant sheep germplasm resources [1], and compared with imported breeds, native Chinese sheep breeds have unique advantages, such as litter size, rough feeding tolerance, and strong stress resistance [2]. Among the native breeds, small-tailed Han sheep are the most common. However, compared with foreign breeds, the meat quality of the small-tailed Han sheep is poor and meat production is not high. Although the imported varieties have good meat quality and high volume, they have poor adaptability to the environment in Northwest China. To retain these advantages of the small-tailed Han sheep and improve its meat quality and production, the small-tailed Han sheep is often used to cross between the female parent and the introduced variety [3], and this crossing displays good heterosis. Dubo sheep [4] and Suffolk sheep are the most commonly introduced male parents. When they are combined with the small-tailed Han sheep for cross-improvement, the hybrid offspring exhibit excellent performance in terms of body weight, carcass weight, and slaughter rate [5] under natural conditions. There are more stocks in the northwest region where the environment is harsh. Although different male parent crossings have obvious effects on improving sheep performance, the genetic mechanisms of these crossings are not clear, and related reports are rare. Therefore, studies on the influence of different male parent crossings on sheep muscle and related regulatory genes are important. The development of transcriptomics has enabled to reveal the mechanism of heterosis in crosses between different male parents.

RNA-sequencing (RNA-Seq) is a recently developed approach for transcriptome profiling that uses deep sequencing technologies. Studies using this new approach have already altered our perception of the complexity of eukaryotic transcriptomes. RNA-Seq also provides a more precise measurement of transcript isoform levels than other methods [6]. In recent years, transcriptome analyses of different breeds of sheep, such as STH sheep [7], Dorper sheep [8], Dorset sheep [9], and Mongolian sheep [10], have been conducted for comparative transcription group analysis. Some important markers for molecular marker-assisted selection (*MAS*) include calpastatin (*CAST*), myostatin (*MSTN*), RB transcriptional corepressor 1 (*RB1*) [11], and fatty-acid-binding protein 4 (*FABP4*) [12]. There are studies on genes regulating muscle growth and development, and some important muscle-regulating genes have also been discovered, such as myogenic differentiation 1 (*MYOD1*), myogenin (*MYOG*), and signal transducer and activator of transcription 3 (*STAT3*) [13,14]. Despite several previous studies on sheep transcriptome analysis and muscle-regulating genes, only a few studies have compared the genetic differences in the sheep transcriptome after crosses between different male parents. Therefore, it is of significance to understand the regulatory mechanisms of different male parent crossings on sheep muscle growth and meat quality from a genetic perspective and to further explore regulatory genes. In this study, a transcriptome comparison analysis of Dubo sheep×small-tailed Han (DP×STH) sheep and Mongolian Suffolk×small-tailed Han (SFK×STH) sheep was conducted to identify important genes that affect sheep meat quality. This research will expand the knowledge base of molecular breeding markers and provide new candidate regulatory factors for future genetic and molecular research to optimize sheep production and meat quality traits.

## MATERIALS AND METHODS

### Animal care

All samples were collected in strict accordance with the code of ethics approved by the Animal Welfare Committee of the College of Animal Science and Technology of Gansu Agricultural University (GSAU-AEW-2017-0308).

### Sample collection and preparation, and production performance measurement

The experimental sheep were selected from Zhangye City, Gansu Province. We selected healthy Dubo sheep and Suffolk sheep (n = 50) and local small-tailed Han sheep (n = 250) with a similar body weight, age, and height to breed naturally at a ratio of 1:5. The DP×STH and SFK×STH crossbred lambs (n = 50) that were healthy, with no significant difference in body weight, and were 7±2 days old when selected. After weaning at 2 months of age, all lambs were randomly assigned to different warm sheds and pens, they were regularly provided with drinking water to ensure similar nutritional levels and feeding management conditions. The experimental period lasted 6 months. From each group, we randomly selected six sheep, which were fasted for 12 h before slaughter; we requested professional butchers to perform neck bloodletting to death on the sheep according to the halal method. Subsequently, the production performance and meat quality of the 12 sheep was measured. Live weight of sheep was measured before slaughter. An electronic scale and a tape measure were used to measure the slaughter performance indicators at 6 months of age, including body weight, body size, and carcass output. We referred to previous studies [15,16] to determine the carcass percentage, slaughter percentage, net meat percentage, and eye muscle area. The quality of the meat was evaluated by shear force, pH, color, and muscle marbling of the longissimus dorsi tissue [17]. The longissimus dorsi were collected, immediately frozen in liquid nitrogen, and stored at −80°C until RNA extraction was completed. The fresh longissimus dorsi muscle was fixed with 10% formaldehyde for 24 to 48 h, conventionally dehydrated, and embedded in paraffin for use in Masson’s trichrome staining.

### Masson’s trichrome staining

In Masson’s trichrome staining, the collagen fibers were stained blue with aniline blue, the muscle fibers were stained red with acid fuchsin and ponceau, red blood cells were stained orange-red, and the nucleus was stained black and blue [18].

The longissimus dorsi was stained using the Masson Trichrome Staining Solution Kit (Solarbio, Beijing, China), according to the manufacturer’s instructions. Briefly, the sections were dewaxed and stained with Weigert’s iron hematoxylin working solution for 5 to 10 min. This was followed by staining with acidic ethanol differentiation solution, rinsing with distilled water for 1 min, staining/rinsing with Masson blue solution for 3 to 5 min, rinsing with distilled water for 1 min, and staining with Ponceau red magenta for 5 to 10 min. Phosphomolybdic acid solution was used to rinse the samples for 1 to 2 min, then washed with a weak acid working solution for 1 min, and finally stained with aniline blue for 1 to 2 min. After staining, the tissues were analyzed using ImageJ software (National Institute of Health, Bethesda, MD, USA).

### Total RNA preparation, cDNA library, and sequencing

The total RNA was extracted from the longissimus dorsi muscle tissue of DP×STH and SFK×STH using TRIzol reagent (TianGen, Beijing, China). A Nanodrop 2000 spectrophotometer and Agilent 2100 bioanalyzer (Agilent Technologies, Santa Clara, CA, USA) were used to evaluate the quality and quantity of RNA. RNA samples with an RNA Integrity Number (RIN) > 8.5 were used for cDNA library construction. Double-stranded cDNA was synthesized using mRNA as a template and purified with the QiaQuick-PCR kit (Qiagen Co. Ltd, Shanghai, China) to obtain the final library. After library construction, a 300-bp fragment was detected to ensure library quality. After testing the quality of the library, the flow pool was used to pool different libraries according to the effective concentration and target offline data volume requirements. After cBOT clustering, the Illumina HiSeq2500 (Illumina, San Diego, CA, USA) high-throughput sequencing platform (HiSeq/MiSeq) was used for sequencing (Sagene Bioinformation Technology Co., Ltd., Guangzhou, China) [19].

### Transcriptome assembly and differentially expressed genes identification

By strict filtering and quality control of the sequencing data (removal of reads containing sequencing adapters, reads with an unknown nucleotide (N) ratio >10%, and low-quality reads with Q≤20), we obtained high-quality reads for the subsequent analysis. These high-quality sequences were mapped to the sheep genome (Oar. v.3.1) using Bowtie2 and TopHat2 (v.2.0.3.12) [20]. The fragments per kilobase million method was used to normalize gene expression levels, which were used to directly compare gene expression differences per million images read [21]. The R software package was used to screen differentially expressed genes (DEGs). The criteria were |log2FC|>1 and false discovery rate (FDR) <0.05. All DEGs were used for gene ontology (GO) term enrichment analysis [22] and Kyoto encyclopedia of genes and genomes (KEGG) pathway enrichment analysis [23]. Pathways with Q<0.01 were considered to be significantly enriched. We focused on the GO terms related to sheep meat quality, as these characteristics are the main factors that affect the performance of domestic animals. Finally, the DEGs enriched in these GO terms were selected as the target DEGs for further analysis [1].

### Gene behavior network analysis targeting differentially expressed genes

The gene behavior network was constructed using Cytoscape software (v.3.7.2), based on the relationships among genes, proteins, and compounds in the KEGG database. A co-expression network was constructed based on the standardized signal strength of the DEGs selected from significant GO terms and KEGG pathways using the Pearson correlation coefficients of candidate DEGs.

### Quantitative real-time polymerase chain reaction assay of the target genes

According to the instructions of the TRANS Reverse Transcription Kit (TransGen Biotech Inc., Beijing, China), complementary DNA (cDNA) was synthesized using the BioTeke Thermo RT Kit (BioTeke, Beijing, China) following the manufacturer’s instructions. RNA (700 ng) was used as a template to synthesize the cDNA with a 20-μL reaction mixture. Quantitative real-time polymerase chain reaction (qPCR) was performed to detect the expression levels of the target genes *MYOD1*, *LOC106990881*, and early growth response 1 (*EGR1*) (qPCR primer sequences are detailed in Supplementary Table S1). qPCR was performed on an ABI 7300 real-time system (Applied Biosystems, Waltham, MA, USA) using a 20-μL reaction mixture containing 1 μL of cDNA. SYBR premix Ex Taq II and specific primers were used for each reaction. The expression of the housekeeping gene *β-actin* was used as an in-group control. A denaturation step was performed for one cycle at 95°C for 30 s. The annealing step was run for 40 cycles at 95°C for 5 s and 60°C for 31 s. All PCR were performed in triplicates. The results were calculated using the 2^−ΔΔCT^ method [1].

### Single nucleotide mutation analysis

Genome analysis toolkit (GATK) (v.3.5) was used to perform variant calling on the transcriptome, and annotation of genetic variants was used to perform SNP/InDel correlation analysis [24].

### Statistical analysis

SPSS Statistics (v.22.0; IBM, Armonk, NY, USA) was used to statistically analyze the production performance and meat quality of DP×STH sheep and SFK×STH sheep populations. Unless otherwise stated, data are expressed as mean±standard deviation. An independent sample *t*-test was used to analyze the production performance, meat quality, and qPCR data. The chart was drawn using OriginPro v.9.1 (OriginLab, Northampton, MA, USA), and results with p≤0.05 were considered statistically significant [25].

## RESULTS

### Production performance comparison

**Figure 1:**
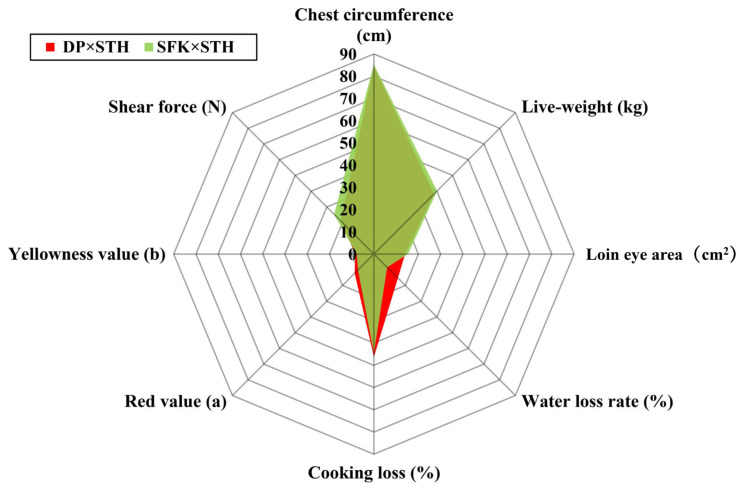
Significantly different indices related to performance and meat quality in the DP×STH and SFK×STH sheep. DP×STH, Dubo sheep×small-tailed Han sheep; SFK×STH, Suffolk×small-tailed Han sheep.

Slaughter performance and meat quality of sheep were measured (Supplementary Table S2 for specific data). The results showed that the yellowness value, redness value, cooking loss, and water loss rate of the DP×STH sheep population was significantly higher than those of the SFK×STH sheep population. The shear force of the DP×STH sheep population was significantly lower than that of the SFK×STH sheep population, which indicates that the meat quality of DP×STH sheep is better than that of SFK×STH. However, the chest circumference, live weight, and loin eye area of the SFK×STH sheep population were significantly or even extremely higher than those of the DP×STH sheep (p≤0.01), which indicates that the production performance of the SFK×STH sheep population was superior to that of the DP×STH sheep (Figure 1).

### Masson staining and analysis

**Figure 2:**
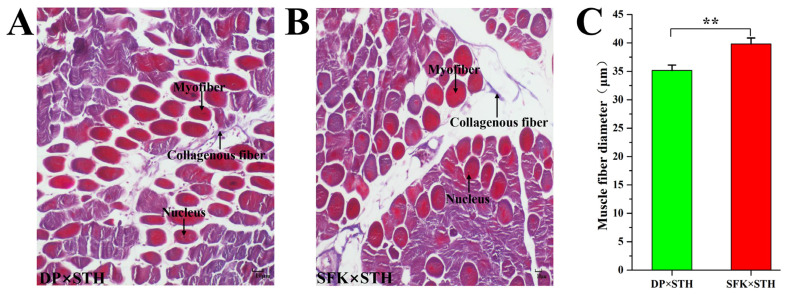
Observation of longissimus dorsi and muscle fiber in DP×STH and SFK×STH sheep (n = 50). (A)–(B) Masson staining of longissimus dorsi muscle. (C) Statistics of single bundle muscle fiber diameter. DP×STH, Dubo sheep×small-tailed Han sheep; SFK×STH, Suffolk×small-tailed Han sheep. ** p<0.01.

To better compare the differences in muscle tissues of the two different male-parent sheep hybrid populations, Masson staining was performed on the longissimus dorsi muscle tissues of DP×STH sheep and SFK×STH sheep (Figure 2A and 2B). The muscle fiber diameter (n = 50) was analyzed. The statistical analysis results showed that the muscle fiber diameter of DP×STH sheep was significantly smaller than that of SFK×STH, indicating that the muscle tenderness of DP×STH sheep, with DP as the male parent, was better than the hybrid with SFK as the male parent. For the SFK×STH sheep, this was consistent with the shear force measured by production performance (Figure 2).

### Transcriptome analysis of longissimus dorsi tissues

**Figure 3:**
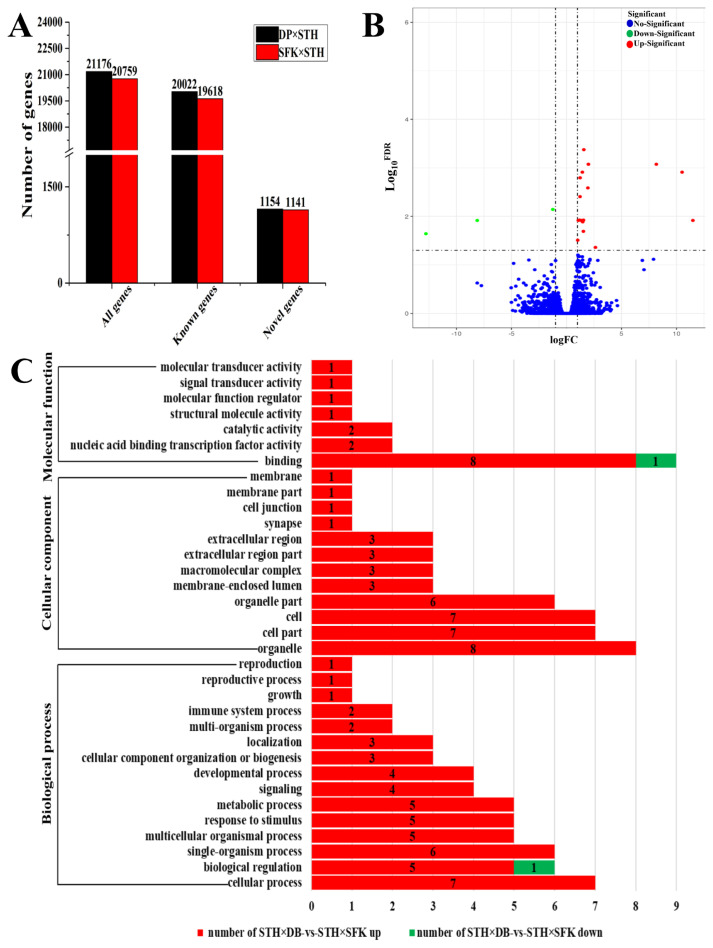
Identification and function analysis of the DEGs from the longissimus dorsi muscle in DP×STH and SFK×STH sheep (n = 6). (A) Total number of gene transcriptome sequences in the two sheep groups. (B) The volcano plot of the DEGs. (C) The GO terms enriched by DEGs in cellular components, molecular function, and biological processes (p<0.05). DEGs, differentially expressed genes; DP×STH, Dubo sheep×small-tailed Han sheep; SFK×STH, Suffolk×small-tailed Han sheep; GO, gene ontology.

By high-throughput sequencing, we obtained an average of 10,336,467,728 bp of clean data in the DP×STH sheep population, and an average of 9,814,835,229 bp in the SFK×STH sheep population. This sequencing data were strictly filtered to eliminate reads containing sequencing adapters, reads with unknown nucleotides (N), and reads of low-quality. Clean, and high-quality data, comprising 10,269,699,032 bp and 9,747,763,445 bp, were obtained for the DP×STH and SFK×STH populations, respectively. After removing the reads containing rRNA, clean reads of 69,215,457 bp and 65,581,840 bp were obtained, respectively. These clean reads were then mapped to the sheep genome (Oar._v.3.1) for transcriptome analysis (Supplementary Table S3). A total of 21,176 genes were annotated in DP×STH sheep (20,022 known genes, 1,154 novel genes), and 20,759 genes were annotated in SFK×STH sheep (19,618 known genes, 1,141 novel genes) (Figure 3A). Using DP×STH sheep as a reference, 22 DEGs were annotated in SFK×STH sheep (genes with FDR<0.05, and log2FC>1 were considered DEGs), including 19 upregulated genes and 3 downregulated genes (Figure 3B; Supplementary Table S4). These DEGs were enriched in 34 GO terms, namely, 15 biological processes, 12 cellular components, and 7 molecular functions (Figure 3C). The KEGG pathway enrichment analysis indicated that the circadian rhythm (ko04710) was a significantly enriched pathway (p<0.05).

### Functional analysis of candidate differentially expressed genes related to meat quality

**Figure 4:**
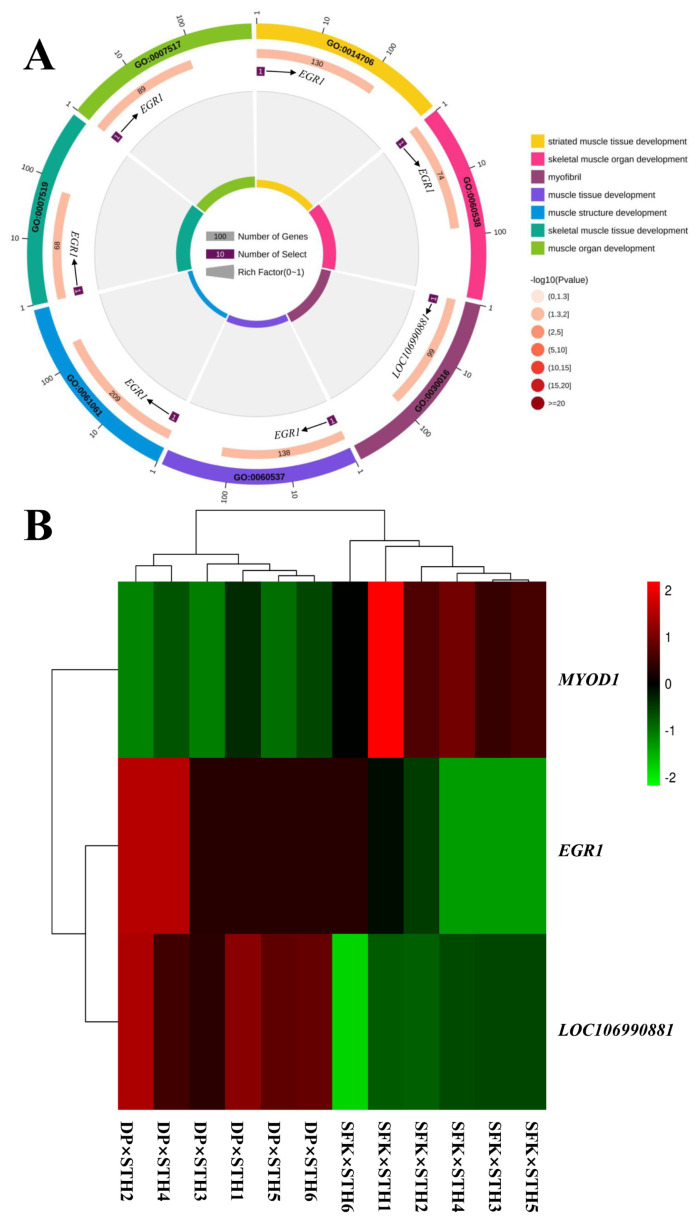
Identifying the candidate DEGs related to meat quality or myofiber in the DP×STH and SFK×STH sheep. (A) Identification and GO term enrichment analysis of the candidate DEGs related to muscle and myofiber in the two sheep breeds. (B) The heat map of the DEGs related to meat quality or myofiber. DEGs, differentially expressed genes; DP×STH, Dubo sheep×small-tailed Han sheep; SFK×STH, Suffolk×small-tailed Han sheep; GO, gene ontology.

In this study, genes related to muscle traits were selected as the target genes, and three upregulated DEGs (*MYOD1*, *LOC106990881*, and *EGR1*) were functionally related to the muscle and muscle fibers. In addition, seven GO terms were found to be closely related to muscle and muscle fibers (Figure 3A). We analyzed the three muscle-related DEGs and the seven muscle-related GO terms and found that *EGR1* was enriched in six GO items (GO:0007519, GO:0060538, GO:0007517, GO:0014706, GO:0060537, GO:0061061), *LOC106990881* was enriched in GO:0030016, and *MYOD1* was not enriched in any of the GO terms (Figure 4A). This finding will require more in-depth research. The heat map showed that there were significant differences between the three meat related DEGs in the two sheep populations, which were crossed from different male parents (Figure 4B).

### Validation of the target genes related to muscle growth and development in the longissimus dorsi

**Figure 5:**
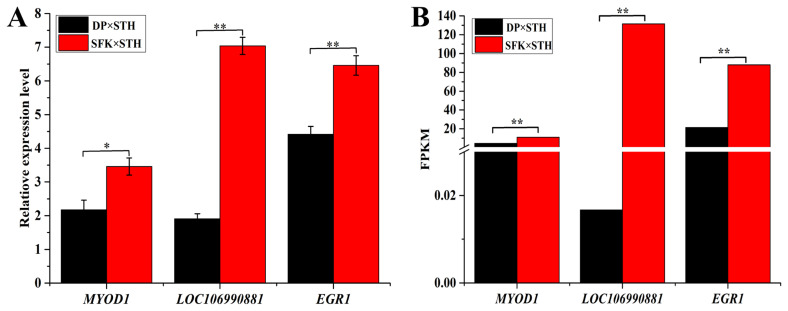
Validation of the target genes in the longissimus dorsi muscle of DP×STH and SFK×STH sheep. (A) The mRNA expression levels of three genes detected by qPCR. (B) Transcriptome analysis detected the mRNA expression levels of three genes. DP×STH, Dubo sheep×small-tailed Han sheep; SFK×STH, Suffolk×small-tailed Han sheep; qPCR, quantitative real-time polymerase chain reaction. * Represents p≤0.05, ** represents p≤0.01.

To evaluate the DEGs identified in the transcriptome analysis, we verified the mRNA levels of the target DEGs related to muscle growth and development. The results showed that, compared with the DP×STH population, the mRNA expression levels of *MYOD1*, *LOC106990881*, and *EGR1* were upregulated in the SFK×STH population. Particularly, the expression of *LOC106990881* and *EGR1* was at least four times higher than that in DP×STH sheep (Figure 5B). The verification results of the qPCR indicated a similar trend (Figure 5A) to the transcriptome analysis results.

### Gene act network-analysis of target differentially expressed genes in sheep muscle

**Figure 6:**
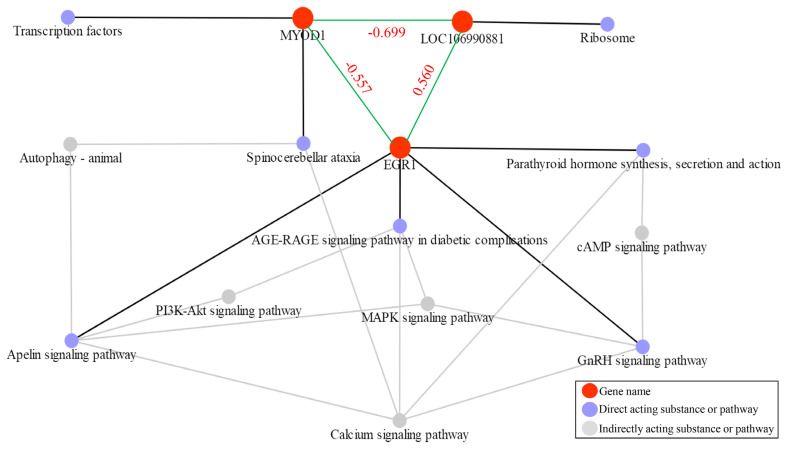
Gene act network analysis of targeted DEGs in sheep meat quality. DEGs, differentially expressed genes.

To further explore the relationship between the target genes, we retrieved the direct or indirect pathways of the three target genes from the KEGG database. We found that *MYOD1* and *EGR1* have a mutual regulatory relationship, whereas *LOC106990881* has a mutual regulatory relationship with *MYOD1* and *EGR1*. However, a regulatory relationship was not discovered. To determine the correlation between these genes, we performed a Pearson correlation analysis on the expression levels of these three genes. We found that the correlation coefficients were r = −0.557 between *MYOD1* and *EGR1*, r = 0.699 between *MYOD1* and *LOC106990881*, and r = 0.560 between *EGR1* and *LOC106990881* (where |r|≥0.8 is a high correlation, 0.5≤|r|<0.8 is a moderate correlation, 0.3≤|r|<0.5 is a low correlation, and |r|<0.3 is irrelevant). This indicates that these three target genes all have a moderately related regulatory relationship (Figure 6).

### Single nucleotide mutation analysis

**Figure 7:**
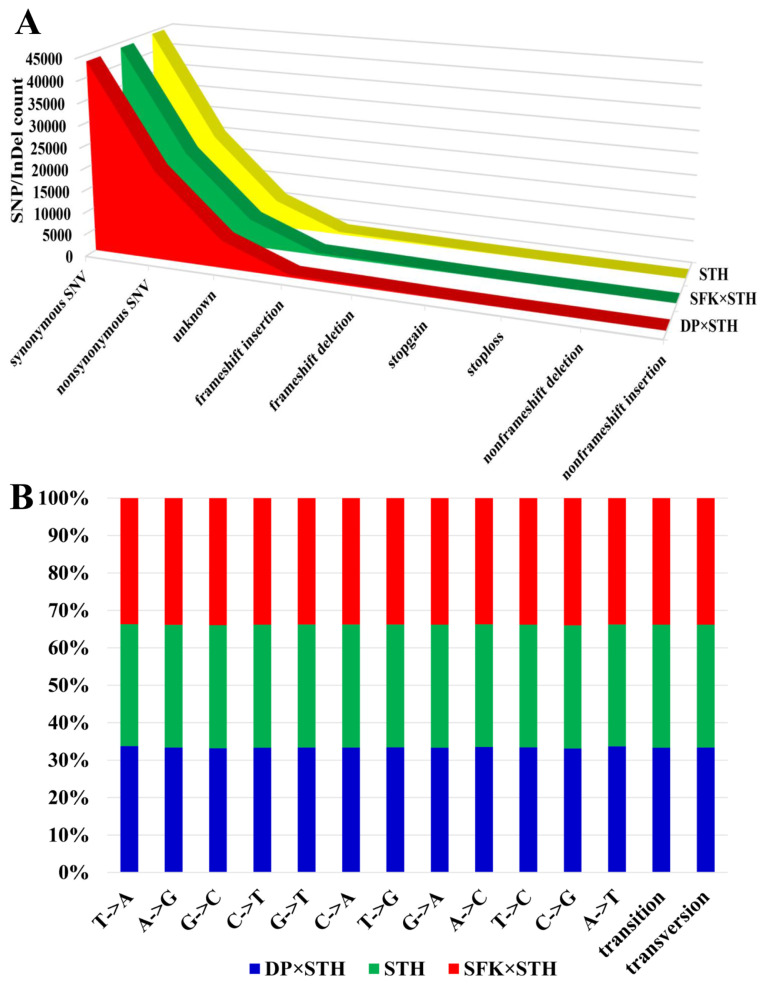
Single nucleotide mutation analysis. (A) SNP/InDel function statistics. (B) SNP mutation statistics. SNP, single nucleotide polymorphism.

The results of the transcriptome analysis showed that the average number of synonymous single nucleotide variants (SNVs) in purebred STH sheep, SFK×STH sheep, and DP× STH sheep populations was 44,351, 43,796, and 43,543, respectively. The average number of nonsynonymous SNVs was 20,186, 20,328, and 20,425, respectively (Figure 7A). There were no significant differences in the number of mutations in the three groups of genes with different functions (Figure 7A) and gene mutation types (Figure 7B). This indicates that the hybridization of different male parents had no effect on gene mutations.

## DISCUSSION

Hybridization is an effective and widely used method to improve livestock production performance and meat quality [26]. People have long recognized the importance of male parent selection to the overall performance of hybrid offspring [27]. However, to date, the genes that affect the quality of sheep meat have not been fully understood. Therefore, it is of significance to study the effects of different male parents and related genes during the crossbreeding of sheep. This study showed that the meat quality of DP×STH sheep is better than that of SFK×STH sheep. The statistical results of Masson’s staining of the longissimus dorsi tissue indicated that the muscle fiber diameter of the longissimus dorsi of DP×STH sheep was significantly smaller than that of the SFK×STH sheep. As early as 2000, it has been pointed out that the muscle fiber diameter is negatively correlated with meat readable [28], that is, the larger the diameter of the muscle fiber, the more tender the muscle, which agrees with the shear force we measured during this study. However, the production performance of the SFK×STH sheep population was better than that of the DP×STH sheep population. This may be because Suffolk sheep are excellent male parents for producing large carcasses, thus the hybrid offspring inherit this favorable trait, which has also been confirmed by another study [29]. The meat quality of Dubo sheep is better, and the hybrid offspring inherit this favorable characteristic [30]; consequently, the meat quality of DP×STH sheep is better than that of SFK×STH sheep.

Less DEGs were detected in the DP×STH and SFK×STH populations in this study compared with the results of previous studies [1,2,31]. Gene production is involved in a close relationship between Suffolk and White-headed Dubo sheep and between Dubo sheep and Suffolk. These are excellent meat breeds [32,33]; therefore, they share similar favorable genes, which are expressed in the hybrid offspring of the male parents. There were a few DEGs. Transcriptome sequencing indicated that the circadian rhythm pathway, specifically the downregulated period circadian regulator 3 (*PER3*) gene, was significantly enriched, indicating that DP×STH sheep possesses strong environmental adaptability traits. This has also been confirmed by a previous study [30]. This may be due to the strong adaptability and cold tolerance of Dorper sheep and small-tailed Han sheep, and the accumulation effect after hybridization, which is also the unique advantage of hybrid sheep [34] Transcriptome sequencing also identified more than 1,100 new genes, which should be further analyzed. In this study, we focused on genes and GO terms related to muscle growth and development. Bioinformatic analysis revealed four GO terms related to muscles and muscle fibers, and two of these four GO terms related to muscle growth and development were enriched. Among the DEGs (*EGR1* and *LOC106990881*), especially *EGR1*, enriched three GO terms related to muscles and muscle fibers, indicating that this gene plays a vital role in muscle growth and development. Previous studies have shown that the transcription level of the *EGR1* gene increases during the differentiation of bovine skeletal muscle cells [35,36], which also supports the findings of this study. The 60S ribosomal protein *L17* (*LOC 106990881*) gene was first discovered in sheep in 2020, and its function has not been annotated or studied. This study shows that *LOC106990881* is closely related to sheep myofibrils, which is important. *MYOD1* belongs to the nuclear protein of the basic helix-loop-helix transcription factor family and myogenic factor subfamily. It regulates muscle cell differentiation by inducing cell cycle arrest, which is a prerequisite for the initiation of myogenesis. The protein encoded by this gene is also involved in muscle regeneration [37]. The effect of *MYOD1* on muscle development has been extensively researched in pigs [38] and cattle [39]. The direct or indirect action pathways or substances of the three target genes were retrieved from the KEGG database. We found that *MYOD1* and *EGR1* have a mutual regulatory relationship, whereas the regulatory relationship between *LOC106990881* and the two aforementioned genes could not be determined. To better understand the correlation between these, we performed Pearson analysis on the expression levels of these three genes and found that the correlation coefficient was r = −0.55 between *MYOD1* and *EGR1*, r = −0.699 between *MYOD1* and *LOC106990881*, and r = 0.560 between *EGR1* and *LOC10 6990881*. This indicates that these three target genes have a moderately related regulatory relationship, which is worthy of a follow-up study to explore and verify this relationship. To explore whether hybridization affects the genes, we analyzed their number and mutation types in the STH, DP×STH, and SFK×STH populations and found that the three groups had different functional gene mutation numbers and gene mutation types. The difference in number was not significant, which meant that the hybridization of different male parents had no effect on gene mutations, but it altered the phenotype by changing the expression of genes in the hybrid offspring. This is also an acceptable explanation for the mechanism of heterosis [40]. In summary, our study confirmed that three DEGs affect the meat quality traits of DP×STH and SFK×STH sheep. However, the role of these candidate genes in selective genetic breeding to improve the performance of sheep and other livestock requires further research.

## CONCLUSION

In this study, we examined the phenotypic differences in the production performance and meat quality of DP×STH sheep and SFK×STH sheep and compared their transcriptomes. The results indicated that the meat quality of DP×STH sheep was significantly better than that of SFK×STH sheep, whereas the production performance of DP×STH sheep was inferior to that of SFK×STH sheep. Finally, we identified three DEGs related to muscle growth and development. These results may improve our understanding of sheep heterosis and molecular marker-assisted selection.

## SUPPLEMENTARY MATERIAL

**Supplementary Table S1**. qPCR primer sequences

**Supplementary Table S2**. Production performance and meat quality differences between the DP×STH and SFK×STH sheep populations

**Supplementary Table S3**. Summary of transcriptomic sequence read mapping in the DP×STH and SFK×STH sheep populations

**Supplementary Table S4**. DEGs of the comparative transcriptome analysis in the DP×STH and SFK×STH sheep populations
