# Supplementary material for: Exploring effects of different male parent crossings on sheep muscles and related regulatory genes using mRNA-Seq
Source: Anim Biosci. 2022 Jan 5;35(8):1129–40. doi: 10.5713/ab.21.0463 (PMC9262716; doi:10.5713/ab.21.0463)
Supplement: Supplementary file 1 [file ab-21-0463-suppl.pdf]

Table S1. qPCR primer sequences

Symbols	sequence 5'-3'	product length
MYOD1	GCTGTTTACTGTGGG<br>CTCGTTGACTTTGCT	107
LOC106990881	CAGAAAACCCCACAA<br>CACACCTGCCAACTC	189
EGR1	CATTCGCATCCACACT<br>CACTCCTGGCAAACTT	152
ACTB	TCCGTGACATCAAGGAGAAGC<br>CAGGAAGGAAGGCTGGAAGA	176

Table S2. Production performance and meat quality differences between the DP×STH and SFK×STH sheep populations

Indexes	DP×STH	SFK×STH	P Value
Body high (cm)	62.17±0.82	63.42±1.50	0.142
Body length (cm)	68.08±1.59	67.42±1.66	0.121
Chest circumference (cm)	84.33±2.71^{b}	85.25±2.96a	＜0.05
Live-weight (kg)	38.26±1.40^{B}	40.15±2.58^{A}	＜0.01
Carcass weight (kg)	19.75±0.54	20.46±1.58	0.096
Net-meat weight (kg)	14.61±0.47	15.14±0.89	0.084
Net-meat percentage (%)	73.97±0.79	74.11±1.94	0.639
Meat bone ratio (%)	38.20±1.20	37.73±1.03	0.861
Dressing percentage (%)	51.64±1.39	50.92±0.96	0.747
Loin eye area (cm2)	14.04±0.97^{b}	15.35±0.96^{a}	＜0.05
Back meat thick (cm）	1.83±0.07	1.81±0.10	0.162
pH	5.46±0.07	5.54±0.08	0.846
Marbling grade	2.50±0.45	2.50±0.55	0.172
Water loss rate (%)	15.28±3.52^{A}	8.40±2.54^{B}	＜0.01
Cooking loss (%)	46.22±0.65^{a}	44.66±2.50^{b}	＜0.05
Brightness value (L)	32.35±0.82	33.97±2.96	0.135
Red value (a)	12.25±1.24^{a}	10.08±1.83^{b}	＜0.05
Yellowness value (b)	8.38±0.97	7.68±1.50	＜0.001
Shear force (N)	21.02±1.46^{b}	25.26±1.65^{a}	＜0.05

a: represent $P < 0.05$, compared with SFK×SHT sheep populations.

Table S3. Summary of Transcriptomic sequence read mapping in DP×STH and SFK×STH sheep populations

Sample	Clean Data (bp)	HQ Clean Data (bp)	Unmapped Pair Reads	Unique Mapped Pair Reads	Mapping Ratio
STH-DP1	10133043226	10078296880	14094019	52157465	78.91%
STH-DP2	9518996061	9466694102	12786469	49651788	79.64%
STH-DP3	10679759447	10607317704	16216403	53549978	76.92%
STH-DP4	11333696707	11242768071	18355042	54738478	75.09%
STH-DP5	10536151244	10463622450	16635716	51794667	75.87%
STH-DP6	9817159683	9759494986	12945119	51003677	79.89%
STH-SFK1	9344370598	9297661542	13295504	47779108	78.37%
STH-SFK2	9009665041	8944580280	11698538	47185388	80.28%
STH-SFK3	10583160640	10507802858	16075381	52258873	76.66%
STH-SFK4	10298328487	10230918080	14837511	52070502	78.04%
STH-SFK5	9838651377	9757854467	15813121	47518955	75.19%
STH-SFK6	9814835229	9747763445	14344011	49362565	77.71%

Table S4. DEGs of the comparative transcriptome analysis in the DP×STH and SFK×STH sheep population

id	STH-DB_count	STH-SFK_count	STH-DB_fpkm	STH-SFK_fpkm	log2(FC)	Pvalue	FDR	significant	Symbol
XLOC_015175	752.8333333	1691.2	5.56	13.45	1.2379	5.38E-07	0.0016	up	TCONS_00047269
XLOC_023254	40.50333333	0	1.648333333	0.001	-8.1105	8.29E-06	0.0121	down	TCONS_00070681
gene11225	2209.666667	4369	21.63333333	46.3	1.0744	9.08E-06	0.0121	up	CILP
gene12155	0	37.558	0.001	2.834	8.1667	1.58E-07	0.0008	up	LOC105615767
gene12670	851.8333333	3049.8	16.77833333	65.282	1.9959	8.42E-08	0.0008	up	CTGF
gene14475	185	528	4.643333333	14.432	1.5755	2.02E-08	0.0004	up	SLC43A2
gene16026	461.3333333	182.2	3.71	1.6	-1.2412	3.44E-06	0.0072	down	PER3
gene19210	193	433	4.516666667	11.004	1.1968	6.86E-06	0.012	up	MYOD1
gene19764	25.16666667	90	0.323333333	1.27	1.9402	9.93E-07	0.0026	up	ABTB2
gene20486	1164	2865.4	8.85	23.336	1.346	8.59E-06	0.0121	up	FAM134B
gene22874	345.155	781.68	10.08	24.8	1.2307	1.69E-06	0.0039	up	CDKN1A
gene22884	72.5	191.8	2.373333333	6.924	1.473	1.12E-05	0.013	up	PIM1
gene2307	0.145	951.662	0.016666667	131.558	11.484	9.79E-06	0.0121	up	LOC106990881
gene25822	2.13	12.954	0.401666667	2.802	2.6295	4.59E-05	0.0437	up	LOC101115593
gene27431	0	197.108	0.001	5.104	10.51	3.30E-07	0.0012	up	LOC101123349
gene5334	218.3333333	632.4	3.23	9.952	1.5358	1.85E-05	0.0204	up	LPIN1
gene56	609	1500.8	7.288333333	19.368	1.4455	3.53E-07	0.0012	up	KLHL30
gene5795	995.95	0	186.1916667	0.001	-12.774	2.18E-05	0.0228	down	LOC105606943
gene6865	94.16666667	227.4	1.168333333	3.03	1.3781	9.87E-06	0.0121	up	NTN4
gene8256	49.66333333	133.318	3.706666667	11.084	1.5447	6.58E-06	0.012	up	LOC105611038
gene9802	718.5	2739.4	21.335	88.04	1.9873	1.62E-07	0.0008	up	EGR1
gene9988	504.6666667	977	1.996666667	4.152	1.0196	3.12E-05	0.031	up	PPARGC1B
